# Machine learning for direct oxygen saturation and hemoglobin concentration assessment using diffuse reflectance spectroscopy

**DOI:** 10.1117/1.JBO.25.11.112905

**Published:** 2020-11-17

**Authors:** Ingemar Fredriksson, Marcus Larsson, Tomas Strömberg

**Affiliations:** a Linköping University, Department of Biomedical Engineering, Linköping, Sweden; b Perimed AB, Stockholm, Sweden

**Keywords:** artificial neural networks, microcirculation, Monte Carlo simulations, multilayer tissue model, diffuse reflectance spectroscopy, hemoglobin oxygen saturation

## Abstract

**Significance:** Diffuse reflectance spectroscopy (DRS) is frequently used to assess oxygen saturation and hemoglobin concentration in living tissue. Methods solving the inverse problem may include time-consuming nonlinear optimization or artificial neural networks (ANN) determining the absorption coefficient one wavelength at a time.

**Aim:** To present an ANN-based method that directly outputs the oxygen saturation and the hemoglobin concentration using the shape of the measured spectra as input.

**Approach:** A probe-based DRS setup with dual source-detector separations in the visible wavelength range was used. ANNs were trained on spectra generated from a three-layer tissue model with oxygen saturation and hemoglobin concentration as target.

**Results:** Modeled evaluation data with realistic measurement noise showed an absolute root-mean-square (RMS) deviation of 5.1% units for oxygen saturation estimation. The relative RMS deviation for hemoglobin concentration was 13%. This accuracy is at least twice as good as our previous nonlinear optimization method. On blood-intralipid phantoms, the RMS deviation from the oxygen saturation derived from partial oxygen pressure measurements was 5.3% and 1.6% in two separate measurement series. Results during brachial occlusion showed expected patterns.

**Conclusions:** The presented method, directly assessing oxygen saturation and hemoglobin concentration, is fast, accurate, and robust to noise.

## 1 Introduction

In optical fiber-based diffuse reflectance spectroscopy (DRS), white light is illuminating tissue, and backscattered light is detected at single or multiple source–detector (s-d) separations. The s-d separations are chosen to allow for contrasting scattering ($\mu_{s}^{\prime }$), absorption ($\mu_{a}$), and tissue geometrical effects. Tissue absorption of light in the visible wavelength range is due to major chromophores, such as hemoglobin, mainly oxyhemoglobin and deoxyhemoglobin. Assessment of microcirculatory hemoglobin oxygen saturation and concentration of blood provides important information of local metabolism and its regulation in health and disease. In skin, epidermal melanin and carotenoids are present affecting foremost short wavelengths,^1^ whereas for higher wavelengths water and lipid absorption is significant.^2^ The calculation of tissue chromophores, which is most often the aim for DRS methods, is commonly done using inverse modeling based on diffusion theory^3^^–^^5^ or Monte Carlo techniques,^6^^,^^7^ where modeled DRS data are fitted to measured DRS data in a nonlinear inverse optimization routine. The most general models are based on Monte Carlo simulations which can be applied to realistic multilayer geometries.^7^ However, the inverse nonlinear search algorithms are time-consuming and may lead to local minima solutions.

Machine learning methods eliminate the need for computationally demanding inverse algorithms. Farrell et al.^3^ demonstrated that an artificial neural network (ANN) can be trained to estimate optical properties (OP), $\mu_{a}$ and $\mu_{s}^{\prime }$, from spatially resolved diffuse reflectance (SRDR) at eight s-d separations. The ANN was trained and evaluated using relative intensities generated with diffusion theory and with added noise, since absolute intensities are more difficult to measure. The evaluation showed that ANN performed twice as good as an inverse algorithm based on nonlinear least-square fitting.

Pfefer et al.^8^ used a fiber-optic probe with six detecting fibers with s-d separations 0.23 to 2.46 mm and a single-layer tissue model with Monte Carlo simulations of SRDR. ANN was applied to estimate $\mu_{a}$ and $\mu_{s}^{\prime }$ in a wide wavelength range (UVA-VIS) for endoscopy applications. They extended the range of OP and added noise in the evaluation data in Ref. 9. Later, they used a similar SRDR probe to estimate $\mu_{a}$ and $\mu_{s}^{\prime }$ in a two-layer model with known top layer thickness.^10^ The estimation was done using an ANN trained on scaled Monte Carlo simulations. They estimated OP for each wavelength and then fitted $\mu_{a}$ for chromophores and $\mu_{s}^{\prime }$ as a standard exponentially decaying function. They comprehensively evaluated their method both theoretically and using physical two-layer phantoms. Despite knowing the top layer thickness, accuracy was moderate.

Chen and Tseng^11^ systematically evaluated any two combinations of SRDR at s-d separations of 1, 2, and 3 mm using an ANN for estimating $\mu_{a}$ and $\mu_{s}^{\prime }$. They trained their ANN with Monte Carlo simulated data without including noise. They found that only SRDR at s-d separations 1 and 2 mm fulfilled their uniqueness of solution criteria. However, their method demands a noise level of <0.5% for both SRDRs, which to our experience is far too low for clinical measurements.

Tsui et al.^12^ proposed using a four-layered tissue model, described by nine parameters that included geometrical and optical properties, for analyzing DRS measurements. An ANN was trained with the nine parameters as input and DRS spectra at three s-d separations as output. This approach, where ANN replaces Monte Carlo in the analysis algorithm, still leaves a time-consuming inverse problem to be solved. In the inverse problem, harmonic generation microscopy determined the thickness parameters, while the other parameters were iteratively determined by fitting modeled DRS data to measurements. Validation simulations, containing 3% random noise, gave chromophore estimation errors below 4%. However, *in vivo* data showed a poor spectral fitting in the 500- to 600-nm wavelength region, likely resulting in erroneous oxygen saturation estimations.

We have previously developed a three-layer skin model for analyzing data acquired using DRS^13^ and DRS integrated with LDF,^7^ using two s-d separations (0.4 and 1.2 mm). An inverse Monte Carlo algorithm was used to fit simulated spectra to measured ones at 32 wavelengths for each s-d separation to directly estimate hemoglobin concentration and oxygen saturation. In contrast to many previous attempts using inverse Monte Carlo or ANN for analyzing DRS data, our algorithm applied spectral constraints on all included chromophores and scattering compounds directly in the inverse algorithm. Hence, no two-stage analysis, where OPs are first estimated separately for each wavelength and chromophore concentrations estimated in a second step, is then needed. This approach benefits from only requiring two s-d separations and a minimal calibration including a dark-, a white-, and a relative-calibration between the two detecting fibers; no calibration on known optical phantoms is needed.

Extensive evaluations of our inverse Monte Carlo algorithm have shown that it is capable of accurately estimating hemoglobin concentration and oxygen saturation,^14^ and that the modeled DRS spectra almost perfectly fit measured DRS data.^15^ However, the inverse Monte Carlo algorithm is computationally demanding which has limited the algorithm to only estimate parameters at rates up to about 2 Hz. It also suffers from the risk of giving erroneous local minima solutions, which has previously been avoided using global search strategies with multiple starting points that further limits the update frequency.

This study aims at investigating if an ANN solution can be used to accurately and robustly estimate RBC (red blood cell) oxygen saturation and the average tissue fraction of RBCs at a rate high enough to capture the full dynamics of a heartbeat. We also aim to investigate if the calibration procedure can be further simplified by leaving out the interchannel intensity calibration. The ANN will be trained on DRS data, simulated using our previously developed and validated skin model. The output parameters (oxygen saturation and RBC tissue fraction) will be estimated directly from the input spectra, without first estimating tissue OP for each wavelength. To enhance the training, instrumentation noise and color drift, mimicking system characteristics, will be accounted for in the training data. The ANN algorithm will be evaluated using Monte Carlo simulated DRS data and measurements from a homogenous intralipid-hemoglobin liquid phantom experiment with varying degree of deoxygenized hemoglobin. Furthermore, results from three *in vivo* measurements during an arterial occlusion release experiment are provided.

## 2 Material and Methods

The principle of the proposed method is to train ANNs with diffuse reflectance spectra from a fiber-based system with two different source–detector distances as input, and red blood cell oxygen saturation and tissue fraction as output. The training data are generated using Monte Carlo simulations of a three-layer skin model with optical and geometrical properties covering a wide range of skin tissue types. A noise model is presented to account for realistic measurement noise in the simulated training data. The approach has many similarities with the method that we have previously presented for multiexposure laser speckle contrast imaging.^16^ The networks are evaluated using an independent set of simulated data from the same type of tissue models, using measurements from intralipid-blood phantoms, and using forearm measurements from an occlusion-release provocation. The results are also compared to a previous method utilizing the same three-layered tissue model and a nonlinear search algorithm for solving the inverse problem.^7^^,^^13^

### 2.1 Measurement System

The measurement instrument used was a Periflux 6000 EPOS system (Perimed AB, Järfälla, Stockholm, Sweden; EPOS is an acronym for enhanced perfusion and oxygen saturation). The system is probe-based and contains both a spectroscopy unit (PF 6060) and a laser Doppler perfusion monitoring unit (PF 6011). In this study, only the data from the spectroscopy unit was used. The probe included one emitting optical fiber connected to a white light source (AvaLight-hal-s-mini, Avantes BV, Apeldoorn, the Netherlands) and two receiving optical fibers placed at a center–center separation of 0.4 and 1.2 mm, respectively, from the light-emitting fiber on the face of the probe. Those two receiving fibers were connected to one spectrometer each (AvaSpec-ULS2048L, Avantes BV). All three fibers were made of fused silica, had a core diameter of 200  μm, and a numerical aperture of 0.37. The system also included a pressure unit (PF 6050) connected to a blood pressure cuff.

In the preprocessing of the spectra, a dark intensity spectrum was subtracted, and white normalization was performed by division with spectra originating from a calibration measurement on a white reference target (WS-2, Avantes BV).

### 2.2 Tissue Model

A three-layer tissue model mimicking skin tissue from an optical and geometrical point of view was used to generate training and evaluation data for the ANN. The model and an efficient method to calculate diffuse reflectance spectra from the model, based on Monte Carlo simulations, have been described previously.^7^^,^^13^ In this study, it was extended with additional free parameters (carotenoids, met-hemoglobin, and different vessel diameters in the two layers). The top layer represents the epidermis, has a variable thickness, and contains melanin and carotenoids but no blood. The second layer represents upper dermis, has a fixed thickness of 0.2 mm, and contains a variable amount of blood of variable oxygen saturation. The third layer represents deep dermis, has an infinite thickness, and contains a variable amount of blood of variable oxygen saturation.

The model is controlled by 15 free parameters. One parameter, $t_{\mathrm{epi}}$, controls the epidermis thickness. One parameter controls the amount of melanin in the epidermis, i.e., the product of $t_{\mathrm{epi}}$ and the fraction of melanin $f_{\mathrm{mel}}$. The absorption coefficient of melanin, based on Jacques,^17^ is calculated as

$$\mu_{a,\mathrm{mel}}(\lambda )=k{\left(\frac{\lambda }{\lambda_{0}}\right)}^{-\beta_{\mathrm{mel}}},$$ (1)

where $k=48.42\;{\mathrm{mm}}^{-1}$, $\lambda_{0}=550\;\mathrm{nm}$, and $\beta_{\mathrm{mel}}$ is a free parameter accounting for the shape of the melanin absorption, dependent on the relative concentration of eumelanin and pheomelanin. The model also contains the carotenoids beta-carotene and lycopene as chromophores in the epidermis, controlled by two parameters, with absorption spectra $\mu_{a,\beta \text{-caro}}(\lambda )$ and $\mu_{a,\text{lyco}}(\lambda )$ as given by Darvin et al.,^18^ having negligible absorption for wavelengths above 550 nm. Thus, the absorption spectrum of epidermis is given as

$$\mu_{a,\mathrm{epi}}(\lambda )=f_{\mathrm{mel}}\mu_{a,\mathrm{mel}}(\lambda )+f_{\beta \text{-caro}}\mu_{a,\beta \text{-caro}}(\lambda )+f_{\text{lyco}}\mu_{a,\text{lyco}}(\lambda ).$$ (2)

One parameter, $f_{\text{blood}}$, controls the average blood fraction in the two dermis layers, whereas one parameter, $r_{\text{blood}}$, controls the difference of the fraction of blood between the layers so that

$$f_{\text{blood},1}=f_{\text{blood}}(1+r_{\text{blood}}),\;\text{and}\;f_{\text{blood},2}=f_{\text{blood}}(1-r_{\text{blood}}),$$ (3)

where $f_{\text{blood},1}$ and $f_{\text{blood},2}$ are the fractions of blood in upper and lower dermis, respectively. In the model, a hematocrit of 43%, a hemoglobin value of 145  g Hb/L blood, and a mean cell hemoglobin concentration of $c_{\mathrm{Hb},\mathrm{RBC}}=345\;g\;\mathrm{Hb}/\text{liter}$ RBC were assumed.

The oxygen saturation of the hemoglobin in the blood in the two layers is calculated from the two variable model parameters $s_{O_{2}}$ and $\Delta_{O_{2}}$ as

$$s_{O_{2},1}=s_{O_{2}}+\Delta_{O_{2}}/2,\;\text{and}\;s_{O_{2},2}=s_{O_{2}}-\Delta_{O_{2}}/2.$$ (4)

The absorption of reduced (deoxygenated) blood, $\mu_{a,\text{red}}(\lambda )$, is based on data from Ref. 19 and for saturated (oxygenated) blood, $\mu_{a,\text{sat}}(\lambda )$, is based on data from Ref. 20. Furthermore, the absorption of methemoglobin is based on Ref. 21. The absorption coefficient of blood is calculated as

$$\mu_{a,\text{blood},n}(\lambda )=(1-f_{\text{met}})[s_{O_{2},n}\mu_{a,\text{sat}}(\lambda )+(1-s_{O_{2},n})\mu_{a,\text{red}}(\lambda )]+f_{\text{met}}\mu_{a,\text{met}}(\lambda ),$$ (5)

where n is the layer number and $f_{\text{met}}$ is the fraction of met-hemoglobin. The absorption spectra of the dermis layers are calculated as

$$\mu_{a,n}(\lambda )=f_{\text{blood},n}c_{\mathrm{vp},n}(\lambda )\mu_{a,\text{blood},n}(\lambda ),$$ (6)

where $c_{\mathrm{vp},n}$ is a vessel packaging compensation factor calculated as^22^^,^^23^

$$c_{\mathrm{vp},n}(\lambda )=\frac{1-\exp[-D_{n}\mu_{a,\text{blood},n}(\lambda )]}{D_{n}\mu_{a,\text{blood},n}(\lambda )},$$ (7)

where $D_{n}$ is the average vessel diameter for layer n, controlled by two parameters ($D_{\mathrm{avg}}$ and $r_{D}$)

$$D_{1}=D_{\mathrm{avg}}(1-r_{D}),\;\text{and}\;D_{2}=D_{\mathrm{avg}}(1+r_{D}).$$ (8)

Three parameters, α, β, and γ, control the reduced scattering coefficient according to

$$\mu_{s}^{\prime }(\lambda )=\alpha [(1-\gamma ){\left(\frac{\lambda }{\lambda_{0}}\right)}^{-\beta }+\gamma {\left(\frac{\lambda }{\lambda_{0}}\right)}^{-4}],$$ (9)

where $\lambda_{0}=600\;\mathrm{nm}$. The reduced scattering coefficient is equal for all three layers. A Henyey–Greenstein phase function with the anisotropy factor set to 0.8 was used.

Monte Carlo simulations were run for various epidermis thicknesses and reduced scattering coefficients for fiber separations corresponding to the probe geometry (0.4 and 1.2 mm). The path-length distributions in each of the three layers were stored for the detected photons. Diffuse reflectance spectra for 28 wavelengths between 475 and 750 nm were calculated for the two fiber separations based on the 15 model parameters. Interpolation was used based on the epidermis thickness and the reduced scattering coefficient for each wavelength, and then the effect of absorption [$\mu_{a,\mathrm{epi}}(\lambda )$ and $\mu_{a,n}(\lambda )$] was added by applying Beer–Lambert’s law for each path-length from the interpolated path-length distributions as described in Ref. 13.

**Fig. 1:**
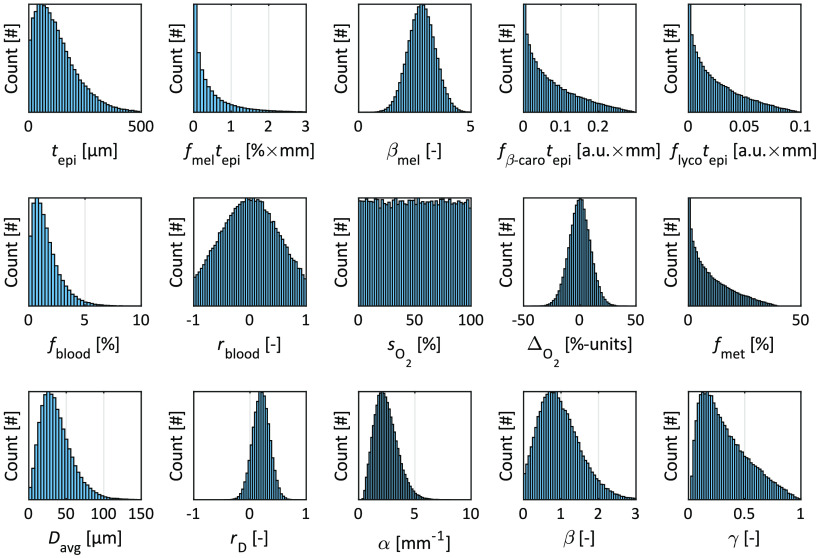
Histograms over the random parameter values for the 15 free model parameters in the training set.

The 15 model parameters were randomly chosen to generate two sets of 100,000 models for training and evaluation of the ANNs. The parameter distributions of the training data set are shown in Fig. 1. The model parameters for the evaluation set were randomly chosen from the same probability distributions.

From each model, the RBC tissue fraction ($f_{\mathrm{RBC}}$) and oxygen saturation in respect to the actual sampling depth were calculated, i.e., the RBC tissue fraction was calculated from the blood tissue fraction with knowledge on the hematocrit of 43% used in the models. The sampling volume was accounted for by calculating the fraction of all optical paths of the detected photons for each of the three layers.

As the hematocrit may vary considerably between individuals and within the circulatory system of a single individual, the tissue fraction of RBC is used as the output parameter rather than the tissue fraction of blood. Another alternative is to present the concentration of hemoglobin, $c_{\mathrm{Hb}}$ [μM=μmole/L tissue], where the conversion is done according to

$$c_{\mathrm{Hb}}=1\times 10^{6}\times \underset{\left[\text{mole}/L\text{ tissue}\right]}{\underset{⏟}{\underset{\left[g\;\mathrm{Hb}/L\text{ tissue}\right]}{\underset{⏟}{\underset{\left[L\;\mathrm{RBC}/L\text{ tissue}\right]}{\underbrace{\frac{f_{\mathrm{RBC}}}{100}}}\times \underset{\left[g\;\mathrm{Hb}/L\;\mathrm{RBC}\right]}{\underset{⏟}{c_{\mathrm{Hb},\mathrm{RBC}}}}}}/\underset{\left[g\;\mathrm{Hb}/\text{mole}\right]}{\underset{⏟}{M_{\mathrm{Hb}}}}}}=f_{\mathrm{RBC}}\times 53.5\;\mu M,$$ (10)

where $c_{\mathrm{Hb},\mathrm{RBC}}=345\;g\;\mathrm{Hb}/L$ RBC according to Sec. 2.2 and $M_{\mathrm{Hb}}=64,500\;g/\text{mole}$ is the molecular weight of hemoglobin.^20^ By multiplying with the oxygen saturation, the concentration of oxygenized and reduced hemoglobin can also be calculated.

### 2.3 Nonlinear Optimization

The proposed ANN-based method was compared to the previous method based on nonlinear optimization of the three-layer model to measured spectra at the two s-d separations.^7^^,^^13^ In that method, the parameters presented in Sec. 2.2 are iteratively updated until the difference between the measured and modeled spectra is minimized. In each iteration, modeled spectra are calculated several times for calculating finite differences, which makes the method relatively time consuming. The relative difference between measured and modeled spectra gives residual spectra, that are used by the optimization routine revealing the quality of the optimization. The nonlinear optimization method used in this study differs from the one presented in Refs. 7 and 13 in the way that wavelengths above 750 nm are not included (up to 850 nm previously), and that all 15 free model parameters in Sec. 2.2 are used (11 parameters previously – no carotenoids, no met-hemoglobin, and same vessel diameter in both dermis layers).

**Fig. 2:**
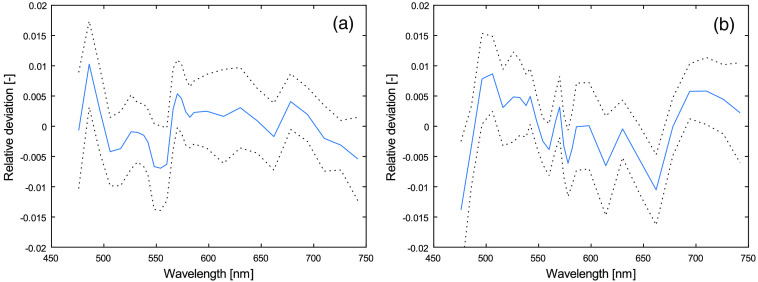
Mean (solid curve) residual ± one standard deviation (dotted curves) for (a) 0.4 and (b) 1.2 mm s-d separations. Residuals are calculated as modeled spectrum divided by measured spectrum minus one.

When using the nonlinear optimization model on measurements on skin, a small systematic residual has been observed, that has not been observed when using the method on modeled spectra or on measurements from liquid phantoms (such as the one described in Sec. 2.7). This systematic residual indicates that the model does not account for all aspects of the tissue that affects the measured spectra. It could, for example, be a geometrical effect not covered by the three-layered model, a missing chromophore, or autofluorescence. The shape of this systematic residual is shown in Fig. 2, based on measurements on the volar side of the forearm on 1557 subjects from a previously published study.^15^^,^^24^ The shape of the residual has no apparent correlation to the level of oxygen saturation or RBC tissue fraction. For the ANN method not to be biased because of this systematic residual, the training and evaluation data is multiplied with a residual model. This model constitutes the multiplication of the intensity of each wavelength of the modeled spectra with a normally distributed random number with mean and standard deviation from Fig. 2. For example, the intensity of 742 nm for the 0.4-mm s-d separation is multiplied with a random number with mean 1−0.005=0.995 and standard deviation 0.007.

### 2.4 Noise Model

The noise characteristics of the spectrometers in the EPOS system was retrieved by performing 100 repetitive measurements on a white reference target (WS-2, Avantes BV). It was observed that the intensity variations per wavelength followed a normal distribution. Thus, the noise of white calibrated spectra can be characterized by a normal distribution with a standard deviation for each wavelength given by the standard deviation of the 100 repetitive measurements divided by their average, i.e.,

$$\eta (\lambda )=\frac{\sigma [w(\lambda )]}{\left\langle w(\lambda )\right\rangle },$$ (11)

where w(λ) is the measurements on the white reference, subtracted with the average dark intensity spectrum. The noise $\eta (\lambda )\xi_{w}(\lambda )$, where $\xi_{w}$ is a random number from a normal distribution with zero mean and standard deviation of unity, was added to the modeled spectra to mimic measurement noise when the raw data was low-pass filtered with a cutoff frequency of ∼4  Hz.

In addition to the sensor noise described above, an uncertainty in color calibration of the spectrometers was added to the spectra. That was done by multiplying the spectra with a straight line with unity mean and a slope s that denoted the relative difference between the value of the line at 475 and 750 nm. The slope $\xi_{\text{slope}}$ was randomly chosen from a normal distribution with unity mean and standard deviation of 0.05.

In total, three noise models affected the modeled training and evaluation spectra $I_{\text{model}}(\lambda )$: the measurement noise $\eta (\lambda )\xi_{w}(\lambda )$, the residual noise $\xi_{\text{residual}}(\lambda )$ from Fig. 2, and the color calibration uncertainty $\xi_{\text{slope}}$:

$$I_{\text{training}}(\lambda )=[I_{\text{model}}(\lambda )+\underset{\text{Measurement noise}}{\underset{⏟}{\eta (\lambda )\xi_{w}(\lambda )}}]\underset{\text{Residual noise}}{\underset{⏟}{\xi_{\text{residual}}(\lambda )}}\underset{\text{Color uncertainty}}{\underset{⏟}{\frac{\lambda -475}{750-475}\xi_{\text{slope}}}}.$$ (12)

### 2.5 Artificial Neural Networks

**Fig. 3:**
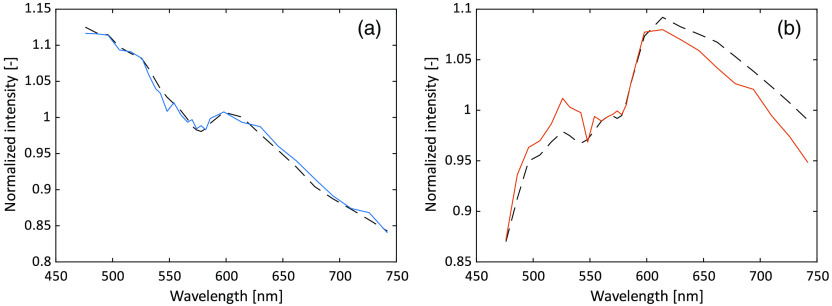
Example of the input data from one model, (a) 0.4-mm s-d separation and (b) 1.2-mm separation. Solid curves represent the model with added noise, dashed without. The target oxygen saturation and RBC tissue fraction were 35% and 0.10%, respectively, in this example.

ANNs were trained using the deep learning toolbox in Matlab 2019b. The ANN:s were small, consisting of a single fully connected hidden layer with 25 nodes and an output layer. The modeled DRS spectra (28 wavelengths at each of the two s-d separations in the general case) were used as input, with or without added noise. The spectra were normalized to their respective mean value prior to input, see Fig. 3 for an example of one of the models used as input. One network was trained with the oxygen saturation in the sampling volume as target, and another with the RBC tissue fraction in the sampling volume as target. The nodes in the hidden layer used the hyperbolic tangent function as activation function. The output layer had a linear activation function for both networks, truncating at 0% and 100% for the oxygen saturation network and at 0% for the RBC tissue fraction network. In the training, Levenberg–Marquardt backpropagation with mean-square-error loss function was used.

Using the default settings, 70% of the data (i.e., 70,000 models) was used for training, whereas 15% was used for validation. When the performance on the validation set decreased during six successive iterations, the training was aborted according to default behavior in Matlab. The final 15% was used to retrieve a performance number that is unbiased to the other 85% of the data. Each network was trained at least 10 times with different initial states, or until the root mean square (RMS) performance, based on the evaluation set, differed <2.5% between the three best networks. The best of those at least 10 trained networks was chosen for further evaluation. This training strategy was tested for various sizes of the hidden layer. No increase in performance could be noted for more than 25 nodes. Therefore, the size of the hidden layer was set to 25 in the networks that were further evaluated.

### 2.6 Data Exclusion

Spectra with a high degree of noise, which is foremost related to a low intensity caused by very high melanin amount ($t_{\mathrm{epi}}\times f_{\mathrm{mel}}$) or high tissue fraction of blood, were excluded from the evaluation. The exclusion criterium was based on the 1.2-mm s-d separation. The difference between two adjacent points in the spectrum (consisting of 28 wavelengths) was calculated in relation to the highest intensity of the two points. If differences above 50% were found for more than two pairs of points, the spectrum was considered too noisy and excluded from the evaluation.

In addition, spectra were excluded from the oxygen saturation evaluation if the hemoglobin signature in the spectra was too weak, generally caused by a very low RBC tissue fraction, as previously described in Refs. 25 and 14.

### 2.7 Tissue Phantom

Liquid phantoms were made from 20% Intralipid with phosphate-buffered saline at a pH of 7.4, as described in Ref. 14. Bovine red blood cells, where plasma was removed by centrifugation, were added to two phantoms with a fraction of red blood cells of about 1.6% and 0.8%, respectively. The phantom was placed in a heated bath with a temperature close to 37°C and stabilized and oxygenated by a magnetic stirrer for 20 min before data collection. The oxygen partial pressure, $pO_{2}$, was measured using a Clark-type electrode. Small batches of dry yeast diluted in water were added to the solution to gradually decrease hemoglobin oxygenation. The $pO_{2}$ was monitored by a voltmeter, calibrated to 160 and 0 mmHg (initial and minimum values, respectively). The expected red blood cell oxygen saturation was calculated from $pO_{2}$ as described in Ref. 14.

When analyzing these measurements, ANN:s were trained without adding the residual model described in Sec. 2.3. Apart from that, the same training data were used.

### 2.8 *In Vivo* Measurements

The method was tested during an occlusion-release provocation on three volunteers. The test subjects were male, aged 24-47 years, and had Fitzpatrick skin types II, III, and V. They were acclimatized in a room holding a temperature of 23°C to 24°C for at least 15 min before the start of the measurement. The measurement started with a 5-min baseline, followed by a 5-min brachial occlusion (200 mmHg), and a 5-min reperfusion phase. The inflation of the blood pressure cuff to 200 mmHg lasted about 10 s, and the deflation to below 50 mmHg lasted about 2 s. The measurement probe was attached using double-adhesive tape (PF 105-1, Perimed AB, Järfälla-Stockholm, Sweden) on the volar side of the right forearm, about 10 cm above the wrist, avoiding any visible vessels. The subjects gave their written informed consent before the start of the measurement, and the protocol was approved by the regional ethical review board in Linköping, d.no. 2018/282-31.

The provocation was chosen to obtain measurement data from large parts of the training space, e.g., both low and high oxygen saturations. Test subjects representing three different skin types were recruited for the same reason.

## 3 Results

### 3.1 Model Evaluation

**Fig. 4:**
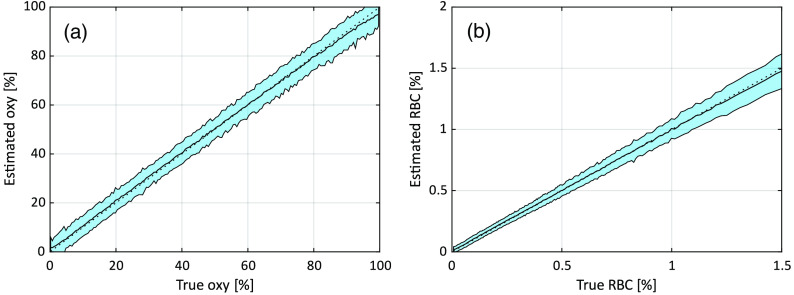
(a) Relation between true and estimated oxygen saturation and (b) RBC tissue fraction for the ANNs trained and evaluated with added noise. The black curves correspond to the average estimated value in each bin, and the shaded areas indicate the standard deviation in each bin. Each bin corresponds to 500 data points from (a) the 85,726 or (b) 97,967 included evaluation models.

The accuracy of the estimated oxygen saturation from the trained ANN:s is expressed as the absolute RMS deviation and coefficient of determination ($R^{2}$) for oxygen saturation, and in addition as the relative RMS for RBC tissue fraction (excluding the lowest 5% since they affect the result considerably). A summary of the results is found in Tables 1 and 2. The column N shows how many of the 100,000 evaluation data models that remained after exclusion as described in Sec. 2.6. Training without noise and including noise in the evaluation data increased the RMS deviations considerably, as compared to no noise in the evaluation data. This shows the importance of including noise in the training data. The fifth row, with noise in both training and evaluation data, is the most relevant result when mimicking a realistic measurement system. Those results are also shown in Fig. 4 where the data are sorted based on the true oxygen saturation or RBC tissue fraction and then grouped in batches of 500 data points. For each group, the average and standard deviation of the estimated oxygen saturation or RBC tissue fraction were calculated. In the figures, the black curve shows the average, and the shaded area represents one standard deviation. The dotted diagonal line represents the ideal case.

**Table 1:** Absolute RMS-deviation and coefficient of determination for estimated oxygen saturation.

Method	Training data	Evaluation data	N	Abs RMS	$R^{2}$
ANN	No noise	No noise	88,009	3.0	0.990
ANN	Noise	No noise	88,009	3.9	0.984
Nonlin. opt.	N/A	No noise	88,009	6.2	0.956
ANN	No noise	Noise	85,726	7.4	0.936
ANN	Noise	Noise	85,726	5.1	0.969
Nonlin. opt.	N/A	Noise	85,726	12	0.844

**Table 2:** Absolute and relative RMS-deviation and coefficient of determination for estimated RBC tissue fraction.

Method	Training data	Evaluation data	N	Abs RMS	Rel RMS	$R^{2}$
ANN	No noise	No noise	99,828	0.056	12	0.984
ANN	Noise	No noise	99,828	0.064	13	0.978
Nonlin. opt.	N/A	No noise	99,828	0.099	27	0.950
ANN	No noise	Noise	97,967	0.12	20	0.922
ANN	Noise	Noise	97,967	0.064	13	0.975
Nonlin. opt.	N/A	Noise	97,967	0.12	26	0.920

The third and sixth rows in Tables 1 and 2 show comparisons to the nonlinear optimization method presented in Sec. 2.3, without and with noise, respectively. The nonlinear optimization method showed approximately twice as high RMS deviations than the respective ANN methods.

In addition to the results shown above, several variants of training data were considered. For example, when only utilizing spectra from one of the s-d separations, there was a negative impact on both oxygen saturation and RBC tissue fraction, largest for the latter. The effect of including the absolute intensity instead of intensity normalized with mean was tested, without any considerable positive effect on accuracy. When adding wavelengths up to 850 nm to the training data, the accuracy increased marginally. These ANNs were trained and evaluated with the same type of noise as described before. The results for these variants are summarized in Table 3.

**Table 3:** RMS deviations and coefficients of determination for other variants of training data.

	Variant	Abs RMS	Rel RMS	$R^{2}$
Oxygen saturation	Table 1, row 5	5.1	—	0.969
	Only long separation	6.2	—	0.954
	Absolute calibration	4.9	—	0.971
	475 to 850 nm	5.0	—	0.971
RBC tissue fraction	Table 2, row 5	0.064	13	0.975
	Only long separation	0.11	19	0.927
	Absolute calibration	0.062	13	0.977
	475 to 850 nm	0.057	12	0.982

### 3.2 Tissue Phantom

**Fig. 5:**
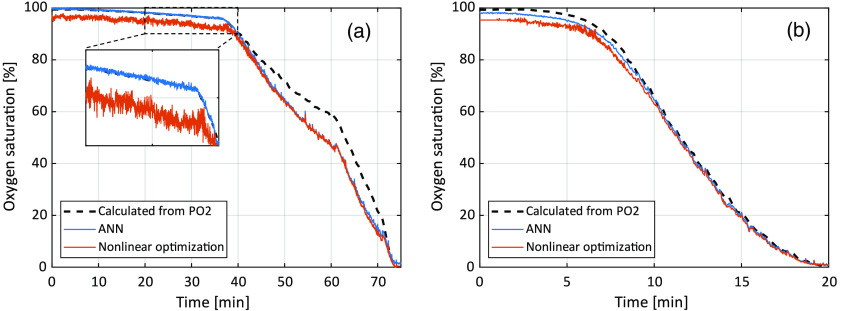
Comparison between the expected oxygen saturation calculated based on measured ${\mathrm{pO}}_{2}$, using the previous nonlinear optimization method, and the new ANN method. Phantom with approximate RBC tissue fraction of 1.6% is shown in (a), and of 0.8% in (b). The inlay in (a) shows the time frame 20 to 40 min with higher resolution.

The proposed ANN method estimated RBC tissue fraction for the liquid phantoms to be 1.4% (1.6% by dilution) and 0.76% (0.8% by dilution), respectively. The estimated oxygen saturation as a function of time is shown in Fig. 5. A comparison to calculated oxygen saturation based on measured $pO_{2}$, as well as to the previous nonlinear optimization method,^14^ is also shown. The absolute RMS deviation between oxygen saturation estimated with the ANN method and calculated from $pO_{2}$ was 5.3 and 1.6 percentage units, respectively, for the two measurements.

### 3.3 *In Vivo* Measurements

**Fig. 6:**
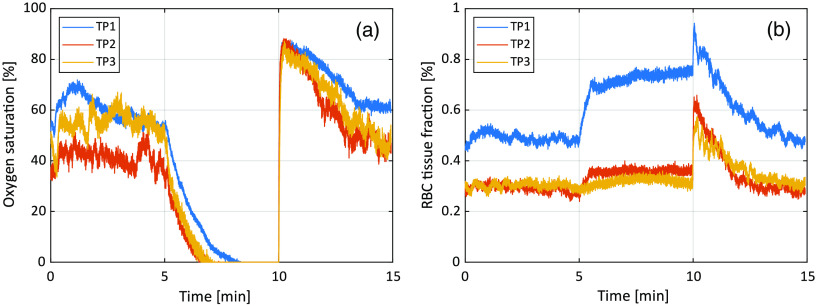
(a) Oxygen saturation and (b) RBC tissue fraction during the occlusion-release test on the three test subjects.

**Fig. 7:**
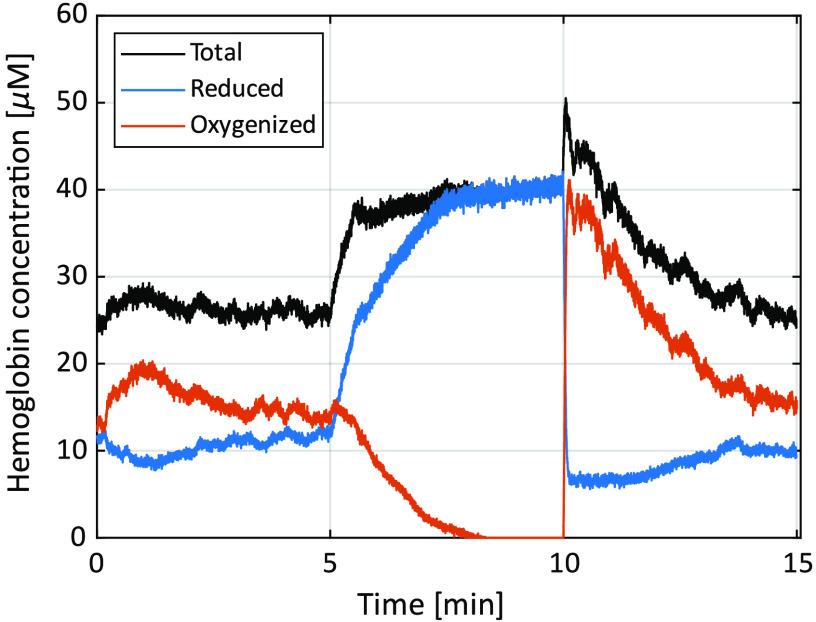
Hemoglobin concentration for reduced, oxygenized and total hemoglobin from TP1.

**Fig. 8:**
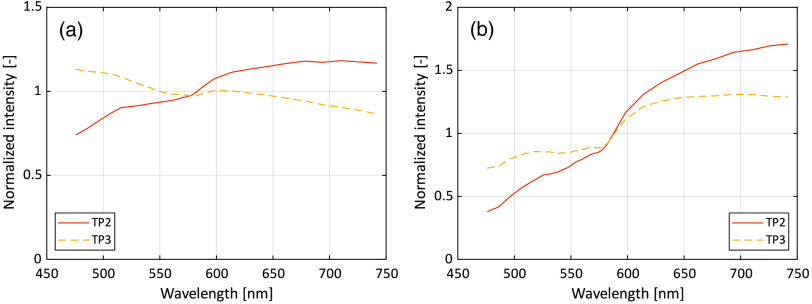
Example DRS spectra at t=16  s in the measurements for TP2 (Fitzpatrick V) and TP3 (Fitzpatrick III). There, they have approximately the same oxygen saturation 38% and RBC tissue fraction (0.30% and 0.26%, respectively). (a) Short (0.4 mm) s-d separation and (b) long (1.2 mm) s-d separation.

The resulting oxygen saturation and RBC tissue fraction from the three *in vivo* measurements are shown in Fig. 6. Test person (TP) 1 had Fitzpatrick skin type II, TP2 had type V, and TP3 type III. RBC tissue fraction and oxygen saturation can be converted to hemoglobin concentration according to Eq. (10). This conversion has been done for TP1 and shown in Fig. 7. A comparison of DRS spectra between TP2 and TP3, at a time point where assessed oxygen saturation and RBC tissue fraction was approximately the same (t=16  s), is shown in Fig. 8.

## 4 Discussion and Conclusion

We have presented a method for estimating oxygen saturation and RBC tissue fraction from recorded DRS spectra, using machine learning with ANNs. The method has higher accuracy and is more stable than the nonlinear search algorithm previously used. It is fast enough to allow for real-time analysis of signals at tens of Hz even on simple embedded CPU’s. The principal difference between the presented method and previous methods where machine learning has been used to solve the inverse problem within DRS^8^^–^^12^ is that the mentioned output parameters are estimated directly from the measured spectra. Previously, this was achieved by first estimating the absorption coefficient wavelength-by-wavelength and then calculating chromophore content (oxygen saturation and RBC tissue fraction) from the absorption spectra. Our direct approach turns out to solve the inverse problem of calculating model parameters from DRS spectra in an effective and stable manner, needing only two s-d separations and a minimal set of calibration measurements. Another key feature that enables our method to fully capitalize on the shape of the DRS spectra is that our training data accurately mimics both the response from a wide range of skin tissue types and system noise.

When dealing with ANNs in any form, the quality of the training data is of utmost importance. First, the training data must be representative to the measurement data, or the results will be inaccurate. In this paper, we propose the generation of training data from a 15-parameter tissue model that is representative of virtually any type of skin tissue, given the parameter ranges in Fig. 1. For other types of tissues, the multilayer model can be adapted, e.g., by excluding the epidermis layer. Some of the parameter ranges are bound to physically possible ranges, such as the oxygen saturation (0% to 100%), while others, such as the scattering parameters, have been chosen so that they widely exceed the ranges that have previously been estimated from thousands of skin measurements.^15^

One of the advantages of using modeled training data is the possibility to quickly generate large amounts of training data covering a wide range of tissue types. It is also the only way to generate tissue relevant training data were the target, i.e., oxygen saturation or RBC tissue fraction, is known with high accuracy. With tissue relevant, we mean inclusion of for example layered structures, vessel geometry (the vessel packaging effect), etc., as well as wide ranges of parameter values as previously discussed. That cannot be obtained using training data from tissue phantoms.

The effect of adding noise to the training data is apparent when studying the results in Tables 1 and 2. The best results are found for the situation when no noise has been added to either the training data or the evaluation data. However, when adding noise to the evaluation data but not to the training data, the results become much worse. Since noise is inevitable in real measurement data, it is evident that the ANNs also need to be trained on data with added noise to retain its performance. In that manner, the networks learn what characterize typical noise and what is spectral information that can be related to output parameters. It is interesting to see that as long as the ANNs are trained with noise, their performance is almost identical regardless if the evaluation data contain noise or not. This “tailored immunity” to noise is visually evident in the zoomed inlay in Fig. 5, where the ANN oxygen saturation fluctuates considerably less than the estimation from the nonlinear optimization method.

Three types of noise are presented in Sec. 2.4: measurement noise, residual noise, and color uncertainty. While the measurement noise and color uncertainty are probably easily grasped, the concept of the residual noise may be more problematic. In the ideal case, there should be no systematic residual between measured spectra and nonlinearly fitted spectra. In our case, the systematic residual is small, generally below 1%, but nevertheless reveals that some detail is missing in the model, potentially an additional geometrical effect, a missing chromophore, or autofluorescence. We have tried a multitude of variants of the model to avoid this, for example, different scattering spectra in the epidermis and dermis, and searched in the literature for other included parameters, without success. Therefore, we decided to include this residual noise. The effect compared to if it was not included in the training data is foremost a slightly increased estimated oxygen saturation at low levels (<10%) of oxygen saturation.

Comparisons are done to the nonlinear optimization method based on the same tissue model, as outlined in Sec. 2.3. The results clearly show that the ANN method is more accurate. It is also several orders of magnitudes faster. The downside is that the ANN method does not reveal how accurate it is for a single measurement, which, however, the nonlinear method does by studying the residual spectra. The ANN method would thus benefit from a technique that could judge if the measured spectra are within the representative range of the spectra in the training data, to avoid erroneous results.

The results presented in this paper for the nonlinear method are slightly worse than previously published.^7^^,^^13^ The reason for this is foremost the noise model that is added to the evaluation data in this study that was not used in the evaluation data in the previous studies. Because the ANN method is faster than the nonlinear optimization method, measurement data will probably be less filtered (less averaged) with the ANN method, resulting in spectra with more noise. The noise level used in this study corresponds to low-pass filtered spectra with a cut-off frequency of 4 Hz, high enough for allowing for studying any changes during a heart cycle, whereas the nonlinear optimization method has been used on data low-pass filtered with 1 Hz or less in previous studies.^15^^,^^24^^–^^26^

A straightforward calibration procedure is proposed, only incorporating dark and white calibration of the spectrometers. No absolute intensity calibration on known phantoms or relative intensity calibration between the two spectroscopy channels are needed, as the recorded spectra are normalized with their own average intensity. This decision is based on the somewhat surprising result that the performance of the method did not substantially increase when taking into account the absolute intensity in the training and evaluation data (see Table 3). This can most likely be explained by how our model can generate training data that fully and accurately captures all variations found in real DRS data from skin tissue. The shape of the two detected spectra is enough for contrasting the estimated parameters. This enables the trained ANN algorithm to fully capitalize on the shape of the measured DRS spectra without needing absolute intensity or the interchannel intensity difference.

The accuracy of the oxygen saturation in the phantom data is on the same level as for the modeled evaluation data. As discussed in the previous paper where the same phantom recordings were used,^14^ the rather large deviation between oxygen saturation estimated by measuring ${\mathrm{pO}}_{2}$ and using the ANN method during the most rapid changes in the first experiment with a rather high concentration of blood, may be due to a decrease in pH or increase in ${\mathrm{pCO}}_{2}$, which alters the relationship between oxygen pressure and oxygen saturation. It may also be due to inhomogeneous mixing of the phantom. If those circumstances would have been more thoroughly controlled, the results would probably had been even better. Nevertheless, these phantom experiments constitute a valuable validation of the ANN method.

The *in vivo* measurements show expected results, with oxygen saturation decreasing to zero during the occlusion phase, and a hyper perfusion leading to oxygen saturation well above baseline at release. The values of both oxygen saturation and RBC tissue fraction during baseline and reperfusion are well in line with previous findings using inverse Monte Carlo.^24^^,^^25^^,^^27^^,^^28^ Differences between the three examples are explained by known spatial and individual variations.^25^

Measured spectra resulting in almost the same oxygen saturation and RBC tissue fraction from TP2 (Fitzpatrick skin type V) and TP3 (type III) are shown in Fig. 8. Notable is the difference in spectral slope between the two persons, whereas the general shape except from the slope is very similar, not at least in the 520- to 590-nm wavelength interval where the dynamics and differences in the hemoglobin absorption spectra is large. The difference in the slope can be explained by a higher melanin concentration in TP2, as the absorption of melanin decreases with wavelength [see Eq. (4)]. It should be noted that even if TP2 had skin type V, the forearm was only moderately pigmented at the time of the measurement as it was performed during the cold and dark time of the year. Thus, further studies are needed to show the feasibility of the proposed method on more pigmented skin.

In this study, we chose only to present ANN results on the estimation of RBC oxygen saturation and tissue fraction, alternatively the hemoglobin concentration which is directly connected to the former two. The presented framework for generating training data is not limited to these parameters. However, we believe that these parameters are of highest clinical value for the end-user. Results from other studies have also shown that ANN methods can demonstrate great results when validated using noise-free simulated DRS data, but when applied to real measurements the accuracy deteriorates.^11^^,^^12^. Hence, before presenting additional parameters estimated using ANN, validation measurements on tissue phantoms constructed to target other parameters are needed.

Once the ANN’s are trained, the calculations are very fast – in the order of microseconds for each set of DRS-spectra. This can be compared to the previous nonlinear optimization method that needed about 0.2 s if a proper starting point was known (such as the solution of the previous time point), and in the order of 1 min if global search had to be used to reduce the risk of local optimum solutions enough.

In conclusion, the proposed approach based on machine learning for estimating oxygen saturation and RBC tissue fraction directly from DRS spectra is more accurate than the previous method based on nonlinear optimization, which in turn has been shown to be superior to an existing state-of-the-art DRS analysis algorithm based on a modified Beer–Lambert’s law expression.^7^^,^^29^ It is several orders of magnitudes faster than the nonlinear optimization method, calculating the output parameters from measured spectra in the order of microseconds using an ordinary CPU. In addition, it is stable in respect to noise and only requires two detecting fibers and a simple calibration. Therefore, the method has great potential to be used in instruments for studying the *in vivo* microvascular status.
